# Development of a community orientation program (COP) as a community-based medical education method for undergraduate medical students: an experience from India

**DOI:** 10.1186/s12909-021-03069-w

**Published:** 2021-12-23

**Authors:** Bayapa Reddy Narapureddy, Shakeer Kahn Patan, C. Sravana Deepthi, Sirshendu Chaudhuri, K. R. John, Chandrasekar Chittooru, Surendra Babu, Khadervali Nagoor, Devika Jeeragyal, Jawahar Basha, Theo Nell, Ravi Shankar Reddy

**Affiliations:** 1 College of Applied Medical Sciences, King Khalid University, Abha, Kingdom of Saudi Arabia; 2 Apollo Institute of Medical Sciences and Research, Chittoor, India; 3 ESIC Medical College, Hyderabad, India; 4 Connect To Grow, Yzerfontein, South Africa

**Keywords:** Community-based medical education, Undergraduate medical education, India

## Abstract

**Background:**

Intra-regional cultural and linguistic differences are common in low- and middle-income countries. To sensitise undergraduate medical students to the social and contextual determinants of health to achieve the ‘health for all’ goal, these countries must focus on innovative teaching methods. The early introduction of a Community Orientation Program (COP) as a Community-based Medical Education (CBME) method could be a game changing strategy. In this paper the methods, evaluation, and implication of the COP in an Indian setting are described.

**Methods:**

The curriculum of the COP was developed based on the analysis, design, development, implementation, and evaluation (ADDIE) model for educational intervention. In this learner-centric and supervised educational program, the key aim was to focus on developing students’ communication skills, observation power and enhancing their motivation for learning through collaborative learning. To meet the objectives of the COP, a situated learning model under the constructivism theory was adopted.

**Results:**

Between 2016 and 2019, 557 students were trained through the COP by visiting more than 1300 households in ten villages. To supplement the students’ observations in the community, more than 150 small group discussions, a health education programme for the community and summary presentations were conducted. The students’ feedback indicated the need to improve the clinical examinations demonstration quality and increase the number of instruments for clinical examinations. More than 80% of students felt that the program would assist them to improve their communication skills, their understanding of the various socio-demographic factors associated with the common diseases, and it will enable them to respect the local culture during their clinical practice.

**Conclusions:**

Early initiation of the COP as a CBME method in the undergraduate medical curriculum in an Indian setting has shown promising results. Further evidence is required to adopt such a program routinely for under-graduate medical teaching in the low- and middle- income settings.

**Supplementary Information:**

The online version contains supplementary material available at 10.1186/s12909-021-03069-w.

## Introduction

Community-based medical education (CBME) is an integral part of the current undergraduate (UG) medical curriculum. It complements classroom and clinical teaching by introducing learning within the community [1–3]. Early introduction of CBME in UG medical education helps the students understand the social determinants of health and the contextual factors associated with various health conditions. It also enhances socio-humanistic skills like communication, collaboration [4–6], listening, observation, leadership, and clinical skills and supports decision-making abilities through group activities [7, 8]. With CBME, the students get the opportunity to be exposed to the community they are going to serve in the future, develop rapport with the community, work as a team, and get the opportunity to learn about various local prevailing diseases [9, 10]. CBME has an inherent ability to identify and adapt to the community’s changing characteristics, like- socio-political situation, environmental condition, and disease trend to remain as a pillar of undergraduate medical teaching.

Undergraduate medical teaching in India is often criticised for its knowledge-based learning rather than focussing on competency-based learning [11–13]. However, the Medical Council of India (MCI) (Now National Medical Commission), the apex regulatory body for medical teaching in India, has restructured the undergraduate curriculum recently to develop core competencies amongst students [14]. The MCI has envisioned that every student should be a clinician, life-long learner, communicator, leader and should act professionally in his/her academic and professional life [14, 15]. The MCI has also emphasised developing key sub-competencies like attitude, ethics, and communication skills amongst the Indian Medical Graduates (IMG) from commencing their undergraduate studies [15, 16]. To meet this changing need in medical teaching, more innovative ideas in medical teaching should be fostered. Although CBME is commonly practised [17], these programs’ experiences are not adequately shared in the scientific literature.

The Community Medicine Department of a private medical college in South India started a CBME program, named the Community Orientation Program (COP) for the first semester students from 2016, which is also the inception year of the college. Since 2018, the COP became a part of the foundation course as laid down by the Graduate Medical Regulations laid down by the MCI. The college, situated in a semi-urban area, is supported by the government district hospital for clinical teaching. The college has a capacity of 150 UG medical students per academic year. The department runs various clinical, academic and research activities in its service area, having more than 0.1 million population. To date, the college has conducted four COPs up until the end of 2019. In this paper, the methods, evaluation, and implication of COP are described.

## Method

The Institutional Curriculum Committee allocated 60 h of training - 30 h theoretical and 30 h practical training - for the Community Medicine Department, as prescribed by the MCI. With inputs from both the Department’s teaching and non-teaching staff, a ‘Community Orientation Program’ (COP) was introduced to complement the classroom teachings. On completion of the COP, students are expected to 1) recognize the community structure, 2) observe the various cultures prevailing in the community, 3) develop communication skills, 4) perform basic clinical examinations, 5) recognize the various determinants of health, 6) identify the locally prevailing clinical conditions, and 6) describe the health needs of the community. Based on the ADDIE (Analysis, design, development, implementation, and evaluation) model for educational intervention, the COP was divided into four stages, namely planning, preparation, implementation, and feedback [18].

### Planning stage

**Fig. 1:**
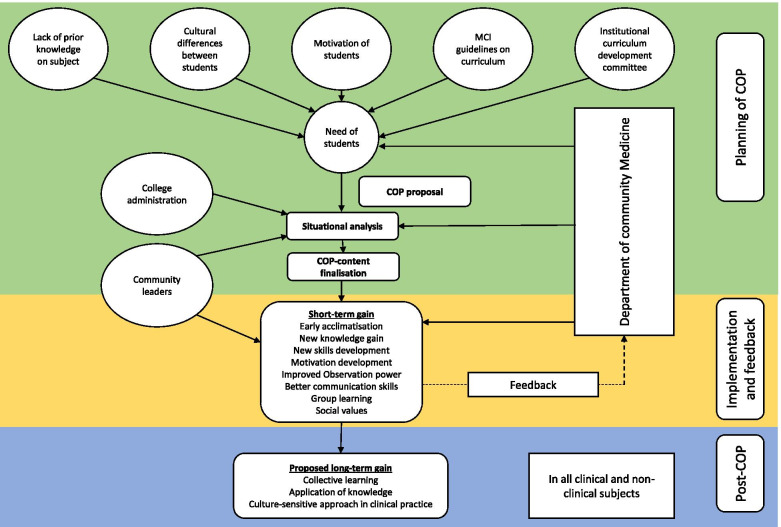
Framework of the Community Orientation Programme, Chittoor, India, 2016–2019

The planning stage consists of the analysis of the students’ background, as well as designing the COP concept. All the stakeholders were also engaged before finalizing the plan. Based on the factors such as students have limited medical knowledge and different motivational level, and unknown teaching environment, students were provided with only the necessary theoretical knowledge before introducing them to the community. (Fig. 1) However, the community described here is fundamentally different to the typical CBME where the learning happens within a community-based clinical setting [2]. For the purposes of this study, community is defined as ‘a group of people (families) living in a defined geographic area with customised cultural practices’. The aim was to enhance the students’ communication skills, observation power, and motivation to learn new things through collaborative learning within a community setting.

The advantages and disadvantages of several adult learning theories were brainstormed, and it was concluded that the situated learning under constructivism theory could be the most appropriate model to deliver on all the objectives [19–23]. Based on the students’ needs, cultural differences, and varying motivational level, a micro-curriculum was prepared to provide structured feedback from the students to restructure the program in subsequent days. (Fig. 1) After multiple discussions with the Institutional Curriculum Committee of the college, the college administration, and the community leaders, the content of the COP was finalized (Table 1).

**Table 1:** Content of Community Orientation Programme for undergraduate students, Chittoor, India, 2016–19

Topic covered	Areas covered	Duration (Hours)	Domain	Level of competency (Miller’s pyramid)	Teaching learning method
Concept on Demography (T)	Basic demographic and fertility indicators	3	Knowledge	Knows	Lecture/ interactive method
Concept of social science and health (T)	Social and cultural factors in health and disease, Structure, and role of family in health in Indian context, socio-economic status classification systems, behavioural psychology	5	Knowledge	Knows	Lecture/ interactive method
Environment and health (T)	Housing standards, waste disposal in community, biomedical waste management	3	Knowledge	Knows	Lecture/ interactive method
National health programs related to common communicable and non-communicable diseases (T)	National Health Policy, National Population Policy, Universal Health Coverage	3	Knowledge	Knows	Lecture/ interactive method
Indian Health System (T)	Structure and function of Indian Health system	1	Knowledge	Knows	Lecture/ interactive method
Demonstration of proforma (T)	How to fill the data collection proforma	4	Knowledge	Knows/ Knows how	Demonstration/ interactive method
Clinical examination (T and P)	Basic clinical examinations	2	Skill	Knows/ Knows how/ Skill/ Shows how	Demonstration
Interview and clinical exam (P)	Interviewing members of allocated families	9	Attitude, skill, communication	Does	Observation
Group discussion (T and P)	On various observations and clinical findings	6	Knowledge, attitude, communication	Knows/ Knows how	Small group teaching/ Peer teaching/ Collaborative teaching
Preparation of health education materials and health education session (T and P)	Diet, lifestyle modifications, and treatment compliance in NCDs	3	Knowledge, Skill, Communication	Knows/ Knows how/ Skill/ Perform independently	Peer teaching/ Collaborative teaching/ interactive method
Data entry/ analysis (P)	–	4	Knowledge, Skill	Knows/ Knows how	Demonstration
Presentation (P)	Theme based	6	Communication/ Skill	Knows/ Knows how/ Skill	Interactive method

*T* Theory, *P* Practical

### Preparatory stage

This stage included planning for the delivering of the educational model. The preparatory stage was divided into two parallel components, namely- 1) the administrative preparation and 2) the academic preparation. A logic model, which summarised the inputs, activities, outputs, and projected outcomes, was prepared (Table 2) to enable a clear understanding of the various indicators of the COP.

**Table 2:** Logic model of the COP

Assumption: There is a need for Undergraduate students to understand the structure of local communities, their culture, and various determinants of locally prevailed diseases.			
Inputs	Activities	Output	Outcome
∙ Teacher’s time ∙ Supporting staff time ∙ Instruments – Sphygmomanometer, weighing scale etc. ∙ Community engagement (Participants time) ∙ Time from local health care workers ∙ Stationaries	∙ Explaining the proforma ∙ Group division ∙ Demonstration of clinical examination ∙ Administrative arrangements ∙ Gantt chart ∙ Mapping of Villages ∙ Sensitization of Community	∙ Home visits by students ∙ History taking ∙ Clinical examination ∙ Group discussion ∙ Establishing referral system ∙ Health education ∙ Data entry and analysis ∙ Presentation in groups	**Short term (1–3 years)** ∙ Understanding theory around disease ∙ Clinical examination ∙ Communication skills in eliciting history ∙ Inter-personnel communication among fellow students **Long-term (4–6 years)** ∙ Improvement in understanding the various obstacles of home-based / community-based treatment of a patient and its solution.
**Impact:** Competent Indian Medical Graduates, Healthy doctor- patient relationship			

#### Administrative preparation

The villages included in the COP were purposefully selected based on the availability of permission from the local authorities and administrative support.

In addition to the teaching and administrative staff, other departments for instance Transport, Food, and Information Technology (IT) were included to ensure the program’s smooth coordination.

#### Academic preparation

Academic preparation includes- social mapping by the social workers with the help of key informants in the village and preparing the interview proforma in English and in local vernacular with instructions for completing the proforma. The proforma is divided into various sections, including- demographic characteristics, socio-economic conditions, environmental conditions (including housing conditions, water, kitchen, and sanitation), health condition (including- the presence of any known disease(s), disability, pregnancy status (if applicable)), and clinical examination of the family members. In 2018, the paper form was replaced with an android-based electronic form. Each year, a nominated senior faculty member divides the other teaching and non-teaching staff’s roles and responsibilities.

### Implementation phase

**Fig. 2:**
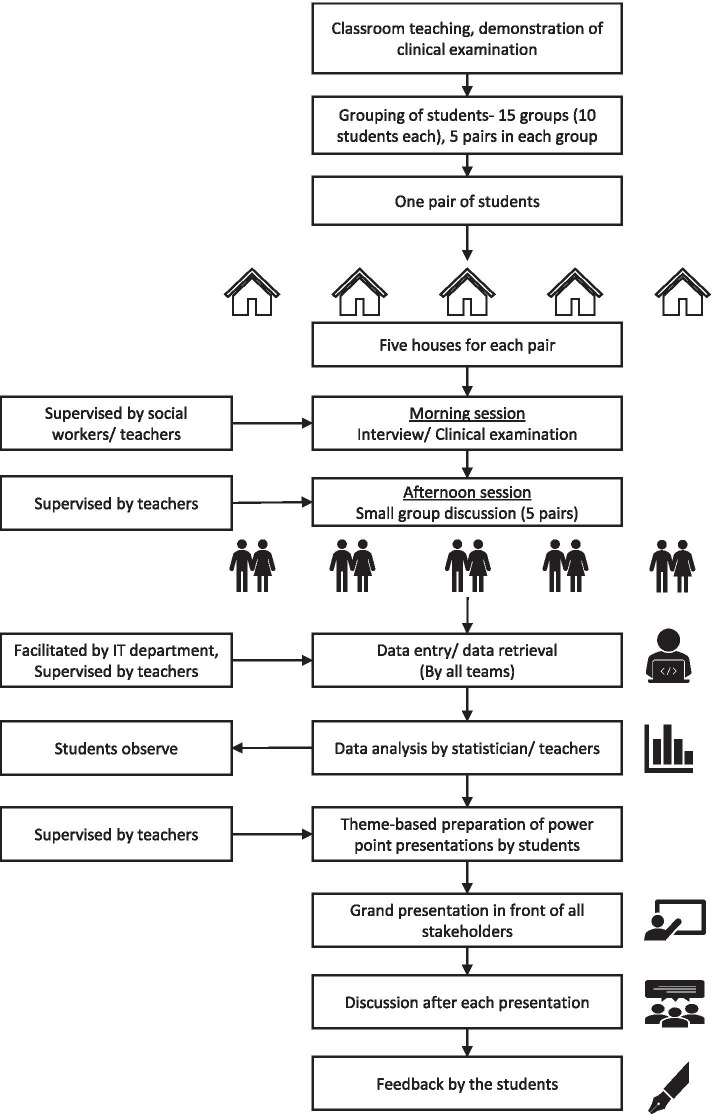
Implementation of the Community Orientation Programme, Chittoor, India, 2016–2019

**Fig. 3:**
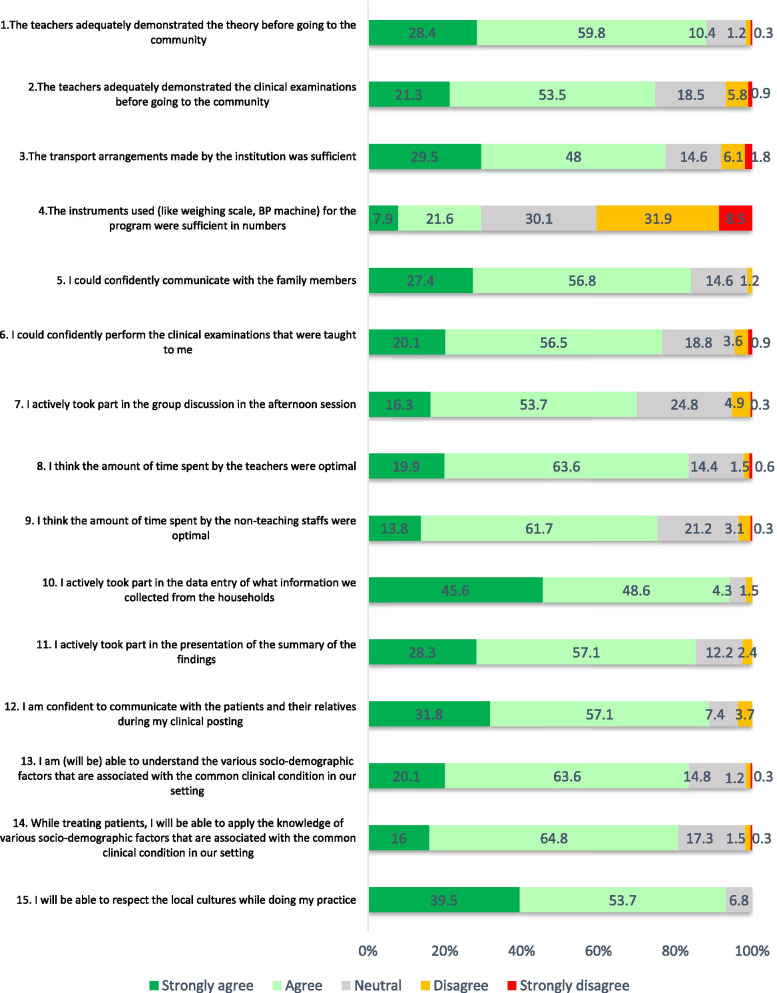
Feedback by the students, Community Orientation Programme, Chittoor, India, 2016–2019 (*n* = 332)

The implementation phase was divided into three distinct phases, namely 1) the theory sessions 2) the field visits (Fig. 2) and 3) data entry, analysis, and presentation of the findings.

#### Theory sessions

These included lectures with interactive sessions facilitated by two to three teachers in each session. In a few sessions like teaching clinical examinations, teachers demonstrated the methods along with the necessary theoretical knowledge. (Table 1) They also explained the field visit process, including the proforma and how to complete it. Students were encouraged to conduct one mock interview with one of their fellow students before visiting the villages to ensure familiarity with the proforma.

#### Field visits

The students were divided into fifteen groups of ten students per group under the supervision of one teacher. Five pairs within each group were pre-assigned, ensuring the availability of at least one student who is well-versed in the local vernacular. Each pair was allocated five houses to interview (Fig. 2).

Each pair conducted the interview sessions with the verbal consent of an adult interviewee of each allocated house. Additionally, they performed the clinical examination and provided health education about a balanced diet, the need for physical exercise, and the importance of regular medical care if someone was already diagnosed with lifestyle disease(s). The social workers and the teachers directly supervised the interview sessions. If required, the teachers ensured an extensive clinical examination and advised on medicines or additional medical care.

During the afternoon small-group sessions, each group of five pairs interacted with each other under the teacher’s guidance. They discussed the various observations made in the community and cleared their doubts by the allocated teacher for the group. The teachers also explained the relationship of various socio-demographic and cultural habits with the diseases they came across during interview sessions. Students were allowed to share their own experiences to help answer questions from their fellow students.

On the final day of the field visit, a selected number of students performed health education activities, which included a role-play and skit at a commonplace in the village to reinforce the health education provided at the family-level during the interview sessions.

##### Data entry and analysis

During 2016 and 2017, students entered data into excel spreadsheets in library computers under the supervision of a statistician and other teachers, facilitated by the IT Department and the library staff. After introducing the electronic questionnaire, the data was retrieved directly from the web and were ready for analysis only with minor corrections. Based on fifteen different themes, the assigned teacher in each group compiled the data on that topic from all 15 groups and analyzed the data; while students observed the analysis. The teachers explained the fundamental analyses (Like- proportion, mean) and interpreting the data.

##### Presentation of findings

Each group presented collective findings on the respective theme using a PowerPoint presentation. Some also used audio-visual modes to explain their observations. Anonymity of all the respondents during the presentations were ensured. After presenting each theme, a group, with their teacher’s aid, clarified questions posted by other groups and teachers. The COP-in-charge summarised the findings at the end of the session. Students were instructed to submit their collated findings to the Department.

### Feedback

A feedback mechanism for the COP through an online questionnaire was created and circulated among the students. The feedback was related to the selected inputs, process, outputs, and outcome indicators described in the logic model.

## Results

### Survey results of the COP

From 2016 to 2019, 557 students enrolled in the program, of which 320 (57.5%) were female. In 2016, the inception year, the input-related lacunae were prominent. (Table 3) After completing the first COP, the team prepared the list of human and non-human resource required, which were partially fulfilled in the subsequent years by the college administration, based on the fund availability.

**Table 3:** Input and output evaluation of Community Orientation Programme, Chittoor, India, 2016–19

Resources utilised	Number/ duration
*Inputs related*	
Teaching staff	Fifteen teachers (Three senior teachers, six junior teachers, four junior resident doctors, two medical officers including one gynaecologist, one statistician). In 2016, there were three junior teachers available.
Non-teaching personnel	∙ Three medical social workers, and one lab technician (Intradepartmental) ∙ Bus drivers: Four in 2016, five from 2017 onwards ∙ Village volunteers: three to five in each COP ∙ Village health workers: three to five in each COP
Instruments	∙ Weighing scale: five in 2016, 15 from 2017 onwards ∙ Sphygmomanometer- five in 2016, 15 from 2017 ∙ Stethoscope: One for each student ∙ Measuring tape: One for each student
Vehicles	Buses: four in 2016, five from 2017
***Output-related***	
Implementation of COP	∙ Number of villages covered: 10 ∙ Number of households covered: 1370 ∙ Number of people covered: 4923 ∙ Number of group discussion in the afternoon sessions: 157 ∙ Number of common health education programs conducted: 10 ∙ Number of groups presented in the final presentation: 57

The students surveyed a total of 4923 people from 1370 households in ten villages till 2019. (Table 2) Out of the population surveyed, 2576 (52.5%) were female, 263 (5.4%) were under five-year-old children, and 559 (11.4%) were older people (> 60 years).

Four hundred and one people had chronic diseases, and fifty were pregnant women. Students could identify chronic diseases including diabetes mellitus, hypertension, physical disabilities (blindness, paralytic conditions due to poliomyelitis, Hansen’s disease, cerebrovascular accidents, and road traffic injuries), mental illnesses, and cancers of various origin. Among the acute cases, acute diarrhoeal and respiratory illnesses among under-five-year-old children. The acute conditions were either treated or referred after clinical examination by the teachers, and students played an observer role.

### Feedback from the students (Fig. 3)

The students indicated us to improve in a few areas during the feedback survey (*n* = 332, response rate 60%). These include- more efforts required from teachers in demonstrating clinical examinations (question 2) and inadequate instruments for clinical examinations. More than 80% of students felt that community exposure through this program would assist them to improve their communication skills, understand the various socio-demographic factors associated with the common diseases, apply the same knowledge in treatment, and will enable them to respect the local culture during their clinical practice (questions 12–15).

## Discussion

In this paper, the methods and results of adopting a relatively new community-based medical education model in an Indian setting, were presented. Students were exposed to learning experiences within their core subject in a real-life situation and in line with the guidelines laid down by the country’s apex educational body. The COP has provided a unique platform for the students to learn based on several complementary learning theories.

The COP has several implications. The students’ introduction to the community helped to get to know one another, which proved to be a critical first step in the bonding process between the two parties. It supported students’ understanding of various social factors, like norms, cultures, behaviours, income and expenditure, hygiene practice of community that indirectly influence the health of the people. This study also supports a similar finding that an early introduction of a CBME in the curriculum grows interest and better understanding of the subject by the students [24–26]. The small group teaching further consolidated their knowledge. A key benefit of the program was development of students’ motivation towards collaborative learning. For example, one pair observed a physically and mentally challenged child within the community. They learned from the family that the child had a rare genetic condition, as informed by their doctor, and the child often suffers from infections. Due to economic constraint, the family faced challenges to meet the child’s medical and social needs. The students also noticed the emotional state of the parents during the interview. In the group discussion, the pair shared their experience with their fellow students. The group learned from the teachers that there are national programs in existence that addresses such conditions. Subsequently, during the grand presentation, students presented a few more interesting clinical cases. Following the presentations, the village administrators and the local health workers were notified to assist in availing government health facilities by the families in need as identified during the program. This experience will enable students to understand the health conditions’ social background, the family’s distress, examine and treat the patient compassionately, and guide the family for further assistance during their clinical postings. In an Indian setting, the doctor-patient relationship is often experienced as difficult [27, 28] and such an educational approach may help in the longer term to improve on that. In addition, the small group teaching, within the ambit of CBME, was found to improve the involvement of the learners and an in-depth interaction between the teacher and the learners [29, 30].

Importantly, as students had limited subject knowledge, it would have been challenging to teach them new medical knowledge. Therefore, the topics were chosen so that only a little understanding of the medical subjects was required and focus on their understanding of social factors that influence the practice of medicine. For example, in the classroom situation, it is taught that ideally, new-born babies should be given only breast milk up to the age of 6 months (exclusive breastfeeding). During the survey, a pair came to know that a new-born baby was given honey and water as pre-lacteal feed instead of breast milk. They shared the experience in the small group session. Another student shared that this is a common practice in her native place as well. With a quality discussion amongst the students and the teacher, the students learned the importance and benefits of breastfeeding and the common myths in different communities. They were also informed about the harmful effects of pre-lacteal feeding. These collaborative learning experiences are not possible in classroom teaching at this early stage of their professional education.

Teaching simple clinical examinations empowered their learning mind. The benefits of the clinical examinations were two-folds. Firstly, the students gained confidence in examining a patient. Secondly, they understood the importance of interpreting their findings. For example, a pair found that a known hypertensive person’s blood pressure was within the normal range. They raised a question based on their classroom learning that blood pressure should be high among hypertensive patients. The teacher then explained the role of medicine and a healthy lifestyle in controlling blood pressure. Learning in such a way enhances their motivation to learn more clinical skills in future. Evidence from integrated educational programmes have shown that early introduction of clinical examination improves the skills during the prospective postings of the medical students [24, 31].

The importance of data was also introduced to the students. Apart from understanding the individual or family-level information, they experienced the importance of interpreting collective data. With simple statistics, they came to know the community’s collective information, which they may not have understood if they would have been exposed only to the family-level observations.

Despite the successes described above, some challenges were experienced. Apart from the administrative challenges of insufficient logistics and instruments described before, academic hurdles were embedded within the teaching-learning process. Teachers need to be continuously trained and retrained to provide quality teaching with the growing change in teaching and learning methods. Although the apex educational body supports teachers’ training [32], the teachers’ adequacy is yet to be evaluated. The objectives of COP may get diminished over time unless horizontally integrated educational approaches support subsequent teaching in other subjects. Additionally, the lack of uniformity in the different universities’ curriculum can reduce such program’s applicability [13]. However, with the introduction of the Graduate Medical Education Regulations, it is expected that these diversified approaches will be addressed in the undergraduate programs. Lastly, the ongoing COVID-19 pandemic reduces the applicability of the COP [33]. Unfortunately, online teaching, a current practice during the pandemic, cannot offer various learning experiences as provided by COP.

## Conclusion

Early initiation of CBME in the UG medical curriculum in an Indian setting shows promising results. The COP is expected to touch upon most of the national- and institutional-level objectives envisioned by the Medical Council of India for the Indian medical graduates. Primarily, students will be able to conceptualize the “health for all” concept and all the citizens’ health rights within this framework of the teaching-learning process. As a part of the foundation course, and within a short time, the holistic approach of the COP showed a potential to facilitate the further learning process of the IMGs by imparting knowledge, attitude, skills, values, responsiveness, and the concept of ethics; and proposes to develop a sensitive and patient-orientated doctor in future. Nevertheless, all stakeholders – academic and non-academic - should collectively find solutions to overcome the various challenges and foster such programs to achieve their goals. Such a promising program can be adopted for UG medical teaching in other Low- and middle- income settings.

## Supplementary Information

**Additional file 1.**

## Data Availability

All data generated or analysed during this study are included in this published article [and its supplementary information files]. The datasets used and/or analysed during the current study available from the corresponding author on reasonable request.

## Abbreviations

CBME: Community-based medical education
COP: Community Orientation Program
IMG: Indian Medical Graduates
IT: Information Technology
MCI: Medical Council of India
UG: Undergraduate

## References

[1] Kelly L, Walters L, Rosenthal D (2014). Community-based medical education: is success a result of meaningful personal learning experiences?. Educ Health.

[2] Mennin S, Petroni-Mennin R (2006). Community-based medical education. Clin Teach.

[3] Worley P, Silagy C, Prideaux D, Newble D, Jones A (2000). The parallel rural community curriculum: an integrated clinical curriculum based in rural general practice. Med Educ.

[4] Dolmans DH, Wolfhagen IH, Heineman E, Scherpbier AJ (2008). Factors adversely affecting student learning in the clinical learning environment: a student perspective. Educ Health (Abingdon).

[5] Dornan T, Littlewood S, Margolis SA, Scherpbier A, Spencer J, Ypinazar V (2006). How can experience in clinical and community settings contribute to early medical education? A BEME systematic review. Med Teach.

[6] Art B, De Roo L, Willems S, De Maeseneer J (2008). An interdisciplinary community diagnosis experience in an undergraduate medical curriculum: development at Ghent University. Acad Med.

[7] Claramita M, Setiawati EP, Kristina TN, Emilia O, van der Vleuten C (2019). Community-based educational design for undergraduate medical education: a grounded theory study. BMC Med Educ.

[8] Jonathan S, Suzanne K, Draper J (2014). Skills for communicating with patients. Emerg Nurse.

[9] Mahoney S, Walters L, Ash J (2012). Urban community based medical education - general practice at the core of a new approach to teaching medical students. Aust Fam Physician.

[10] Strasser RP (2010). Community engagement: a key to successful rural clinical education. Rural Remote Health.

[11] Anshu SA (2016). Evolution of medical education in India: the impact of colonialism. J Postgrad Med.

[12] Chacko TV (2013). Improving quality of medical education in India: the need to value and recognize academic scholarship. J Pharmacol Pharmacother.

[13] Shah SR, Ahmed R, Munir M, Masood S, Aijaz Y, Manji AA-K (2017). Medical education system in South Asia and its consequences on our health: a review. JCDR..

[14] Medical Council of India (2018). Competency Based Undergraduate Medical Curriculum for the Indian Medical Graduate.

[15] Kulkarni P, Pushpalatha K, Bhat D (2019). Medical education in India: past, present, and future. APIK J Intern Med.

[16] Medical Council of India (2018). Attitude, Ethics Commun.

[17] Talaat W, Zahra L (2014). Community based education in health professions global perspectives.

[18] Almomen RK, Kaufman D, Alotaibi H, Al-Rowais NA, Albeik M, Albattal SM (2016). Applying the ADDIE—analysis, design, development, implementation and evaluation—instructional design model to continuing professional development for primary care physicians in Saudi Arabia. Int J Clin Med.

[19] Abela J (2009). Adult learning theories and medical education: a review. Malta Med J.

[20] Badyal DK, Singh T (2017). Learning theories: the basics to learn in medical education. Int J Appl Basic Med Res.

[21] Torre DM, Daley BJ, Sebastian JL, Elnicki DM (2006). Overview of current learning theories for medical educators. Am J Med.

[22] McGrath V. Reviewing the evidence on how adult students learn: an examination of Knowles’ model of andragogy. Adult Learner. 2009; Available from: https://eric.ed.gov/?id=EJ860562 [cited 23 Sep 2020].

[23] Artemeva N, Rachul C, O’Brien B, Varpio L (2017). Situated learning in medical education. Acad Med.

[24] Vyas R, Jacob M, Faith M, Isaac B, Rabi S, Sathishkumar S (2008). An effective integrated learning programme in the first year of the medical course. Natl Med J India.

[25] Sathishkumar S, Thomas N, Tharion E, Neelakantan N, Vyas R (2007). Attitude of medical students towards early clinical exposure in learning endocrine physiology. BMC Med Educ.

[26] Rawekar A, Jagzape A, Srivastava T, Gotarkar S (2016). Skill learning through early clinical exposure: an experience of Indian medical school. J Clin Diagn Res.

[27] Kar SP (2017). Addressing underlying causes of violence against doctors in India. Lancet.

[28] Bawaskar HS (2014). Violence against doctors in India. Lancet.

[29] Pal R, Kar S, Zaman FA, Jha DK, Pal S (2012). Assessment of impact of small group teaching among students in community medicine. Indian J Community Med.

[30] Burgess A, van Diggele C, Roberts C, Mellis C (2020). Facilitating small group learning in the health professions. BMC Med Educ.

[31] Krishnan A, Misra P, Rai SK, Gupta SK, Pandav CS (2014). Teaching community medicine to medical undergraduates-learning by doing: our experience of rural posting at all India Institute of Medical Sciences, New Delhi India. Natl Med J India.

[32] Mitra J, Saha I (2016). Attitude and communication module in medical curriculum: rationality and challenges. Indian J Public Health.

[33] Rose S (2020). Medical student education in the time of COVID-19. JAMA..

